# Difficulties of Bariatric Surgery after Abdominoplasty

**DOI:** 10.1155/2014/620175

**Published:** 2014-11-06

**Authors:** Bora Karip, Hasan Altun, Yalın İşcan, Martin Bazan, Kafkas Çelik, Yetkin Özcabı, Birol Ağca, Kemal Memişoğlu

**Affiliations:** ^1^Department of General Surgery, Fatih Sultan Mehmet Training and Research Hospital, 34752 Istanbul, Turkey; ^2^Department of General Surgery, Liv Hospital, 34340 Istanbul, Turkey; ^3^Bariatric and Metabolic Institute, Cleveland Clinic, Cleveland, OH 44195, USA

## Abstract

During laparoscopy, the main problems of patients who have undergone previous abdominoplasty are inadequate pneumoperitoneum secondary to fibrosis and reconstructed anatomic landmarks for trocar placement. In this study, we present our laparoscopic bariatric experience in two patients with previous abdominoplasty. The procedures were a laparoscopic sleeve gastrectomy and a robotic Roux-en-Y gastric bypass. Both operations were done successfully by an abdominal wall traction technique, cutting fibrotic tissue and choosing new landmarks. We conclude that after abdominoplasty bariatric surgery can be performed safely either using conventional laparoscopic technique or robotically.

## 1. Introduction

Laparoscopic abdominal surgery is challenging in patients who have had a previous abdominoplasty surgery. The main problems which may occur during laparoscopy are inadequate pneumoperitoneum secondary to fibrosis of abdominal wall and reconstructed anatomic landmarks for trocar placement. About one-third of bariatric surgery patients require a body shaping procedure after massive weight loss [1]. These patients are also candidates for revisional bariatric surgery. In this study, we aim to share our experience in revisional bariatric surgery among two patients with a history of previous abdominoplasty.

## 2. Case Presentation

### 2.1. Case  1

A 38-year-old female patient with a history of adjustable gastric banding performed four years ago was admitted to our clinic. Two years after banding, the band had been removed due to slippage and a simultaneous abdominoplasty had been performed. The patient regained weight, her BMI was 43.2 kg/m² at time of admission, and a laparoscopic sleeve gastrectomy (LSG) was planned. Due to the reconstructed localization of umbilicus, we used the xiphoid bone as a landmark for the insertion of the first trocar. 20 cm below the xiphoid cartilage, a 15 mm transverse skin incision was made 2 cm left of the midline.

**Figure 1:**
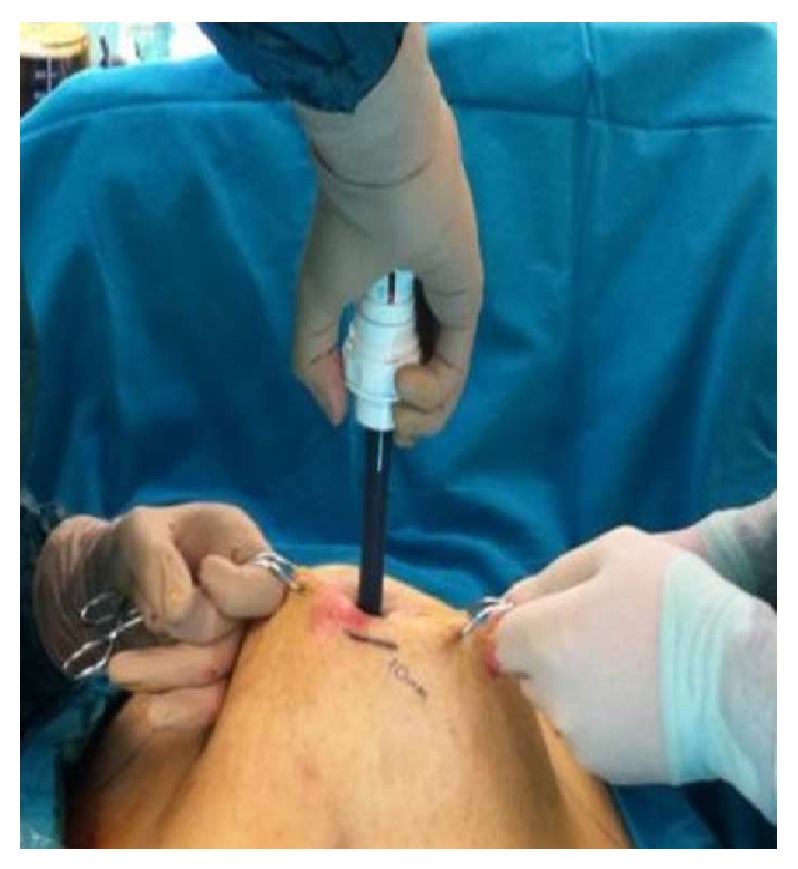
Counter traction of the skin edges during trocar insertion.

After that, two edges of the incision were pulled upwards by towel clamps and a bladed trocar (Versaport Plus manufactured by Tyco Healthcare) was inserted with direct insertion technique through the fascia (Figure 1). Secondary to the fibrosis of subcutaneous tissue, pneumoperitoneum was insufficient. Abdominal pressure reached 14 mmHg after 2 liters of carbon dioxide insufflations. Additional trocars were located by using the same towel clamp pull technique under direct vision. Trocar sites were chosen superior than our routine in the light of arcus costarum rather than the neoumbilicus. A standard sleeve gastrectomy was performed and the gastrectomy specimen was removed from the abdomen without any difficulty. On the 3rd postoperative day, patient was discharged.

### 2.2. Case  2

A 44-year-old female patient with type 2 diabetes mellitus was admitted to our clinic for robotic gastric bypass. Her BMI was 35.6 kg/m². She had a history of a LSG 5 years ago and an abdominoplasty two years after LSG. She regained weight in the last year. As mentioned in the first case, the same attempt was carried out; however the trocar blade could not perforate the fibrosis. The fibrotic tissue was then perforated by a number 11 scalpel and this gate was enlarged by scissors to create a 1 cm free space. Then an 11 mm trocar could be placed with the method as described earlier. Abdominal wall compliance was very low, and pneumoperitoneum could be established with 1.8 liters of carbon dioxide. Additional robotic trocars could be placed in the left subcostal area again by hanging the trocar sites with towel clamps to avoid possible intestinal injury. It was difficult but possible to reach the ligament of Treitz and then to divide the great omentum. A standard gastric bypass was performed successfully. Her postoperative course was uneventful and she was discharged 4 days after surgery.

## 3. Discussion

Unsuccessful bariatric procedures are frequent in the morbidly obese population. The causes of weight regain are multifactorial and related to patient- and procedure-specific factors [2]. One-third of postbariatric surgery patients achieve massive weight loss necessitating reconstructive body contour surgery [1]. When a revisional bariatric procedure is required, this previous cosmetic surgery may lead to some problems. The limited publications about this problematic group of patients have underlined the importance of fibrosis secondary to wide dissection of abdominoplasty. Sometimes creation of a pneumoperitoneum can be impossible because of the thickened abdominal wall [3]. Among laparoscopic colorectal surgery patients who had previously undergone abdominoplasty surgery, the main technical problem was found to be the loss of abdominal wall compliance. Atallah et al. [4] also found the aesthetic results of laparoscopy acceptable. Some alternative methods for laparoscopic trocar placement were described for patients following transverse rectus abdominis myocutaneous flap reconstruction. For this group of patients, great care should be given to avoid injury to blood supply of the flap [5, 6].

Some authors offer an open Hasson technique and its modifications for initial access [7]. Direct trocar insertion is found to be a safe technique in laparoscopic surgery. The primary trocar is placed without prior insufflation. This technique avoids the risks associated with Veress needle but it may increase the risk of major vessel injury [8, 9]. The main problem encountered during abdominoplasty patients seems to be perforation of fibrotic tissue. This problem could be overcome by counter traction with towel clamps and perforation of fibrosis with a scalpel in one of our cases. After abdominoplasty, the anatomic points for trocar placement changed [5]. The umbilicus is the preferred anatomic landmark to determine midline, but after abdominoplasty reconstructed umbilicus may be relocated left or right side of its original location. For initial access, a constant landmark like the xiphoid bone or left subcostal area (Palmer's point) may be more useful than a replaced umbilicus.

**Figure 2:**
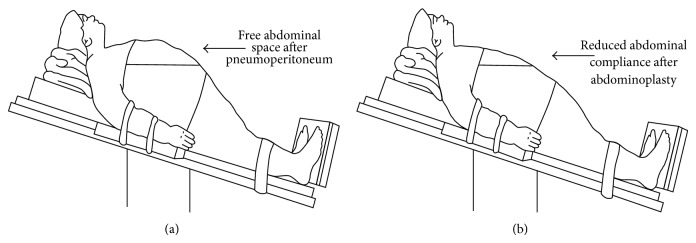
(a) Figure demonstrating the free abdominal space after pneumoperitoneum in reverse Trendelenburg position. (b) Reduced abdominal compliance after abdominoplasty.

As demonstrated in Figure 2, free space in the abdomen is reduced after abdominoplasty. Upper parts of the abdomen can achieve more free space for laparoscopic instruments, especially in reverse Trendelenburg position which is essential for laparoscopic upper gastrointestinal surgery. To gain further abdominal space, reverse Trendelenburg with flexing of the legs at the hips should be tried [10]. Therefore, we used more superior trocar sites when compared to routine placement.

Previous abdominoplasty is not a contraindication for bariatric surgery. Reconstructed anatomy, fibrosis, and lower abdominal compliance seem to be the main problems. Choosing proper trocar sites using stable landmark as guidance and counter traction of skin during trocar access seem to solve these problems.
